# Supplementary material for: Spatial confidence sets for raw effect size images
Source: Neuroimage. 2019 Dec;203:116187. doi: 10.1016/j.neuroimage.2019.116187 (PMC6854455; doi:10.1016/j.neuroimage.2019.116187)
Supplement: Multimedia component 1 [file mmc1.pdf]

Appendix A. Supplementary Human Connectome Project Results

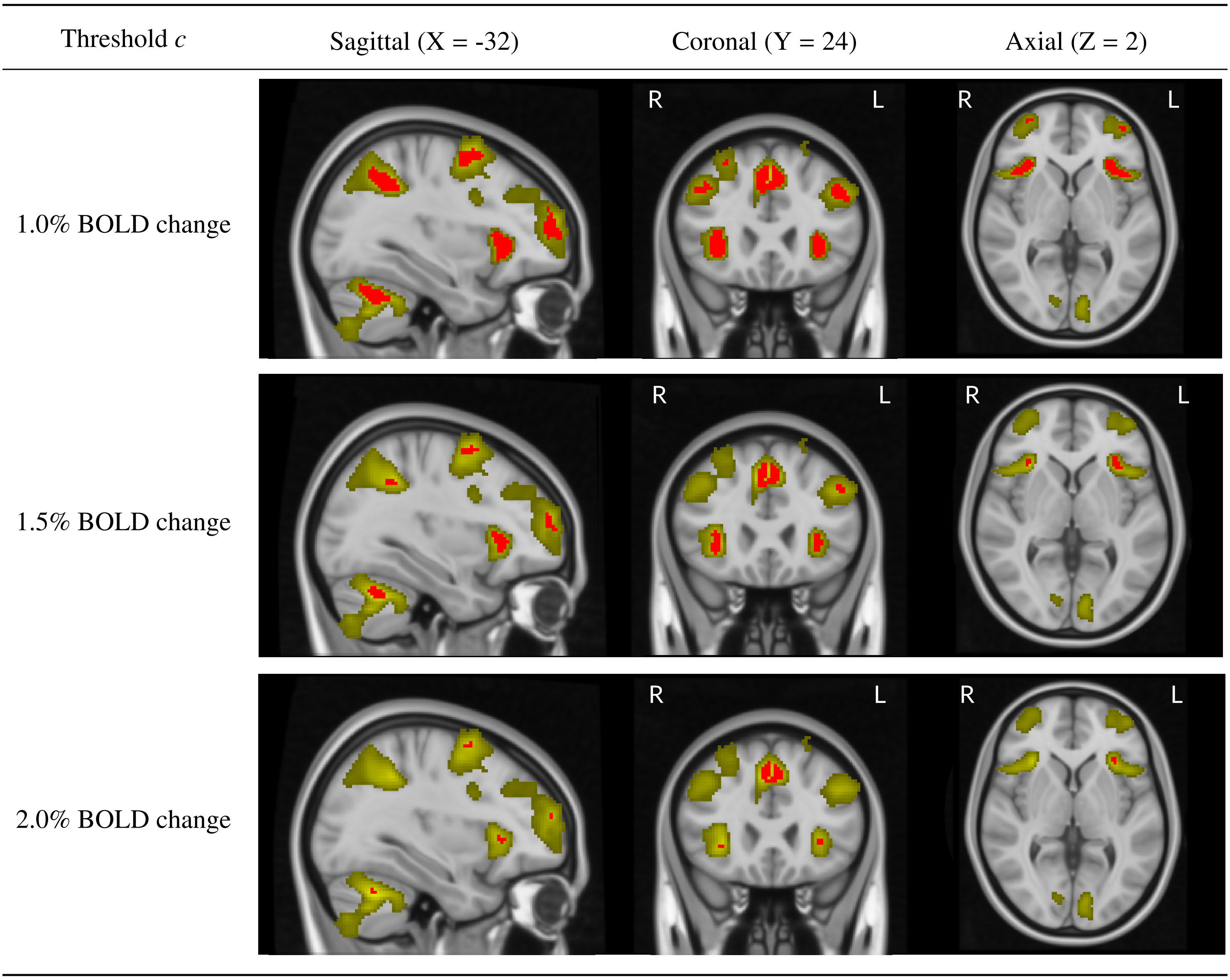

Supplementary Figure 1: Comparing the upper Confidences Sets for the HCP working memory task data (same slice views as Fig. 15) with the thresholded t-statistic results obtained by applying a traditional group-level one-sample t-test, voxelwise $p < 0.05$ FWE correction (green-yellow voxels). While over 25,000 voxels were determined as statistically significant with the standard inference method, less than 5,000 voxels were asserted to have at least a 1.0% BOLD change by the CSs. In particular, the two statistically significant clusters spanning the left and right side of the frontal lobe contained almost no voxels with a practical effect size of over 1.5% BOLD change.

Threshold c	Sagittal (X = -4)	Coronal (Y = 16)	Axial (Z = 48)
1.0% BOLD change	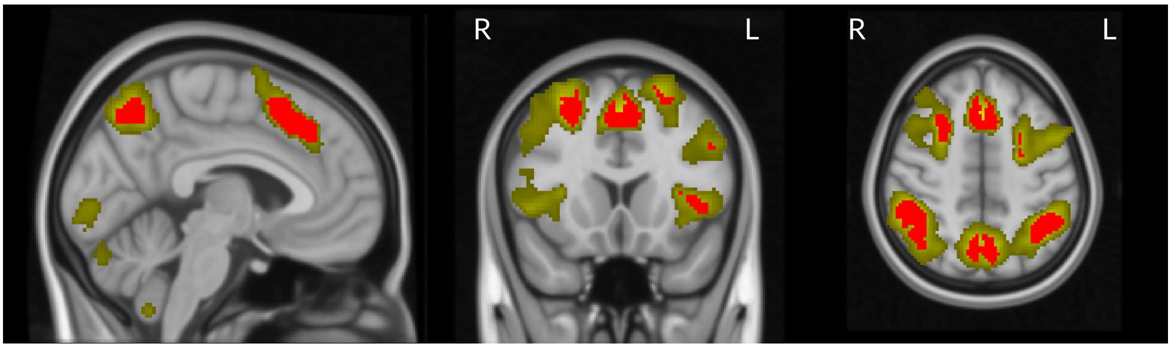		
1.5% BOLD change	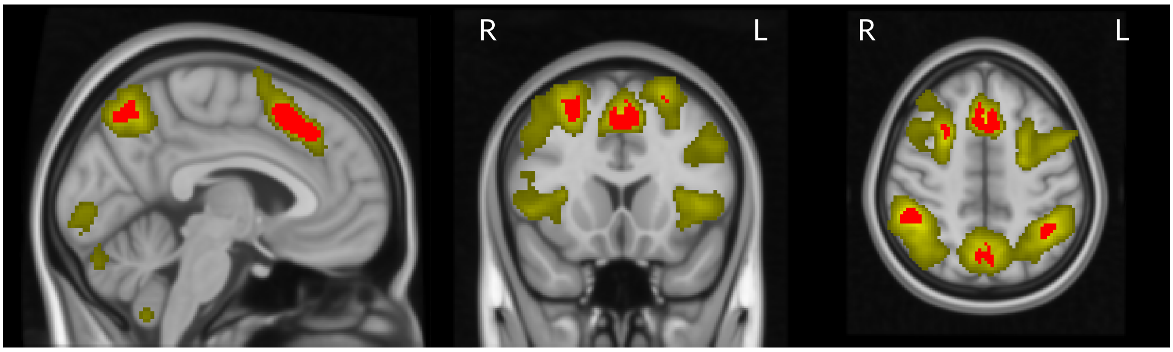		
2.0% BOLD change	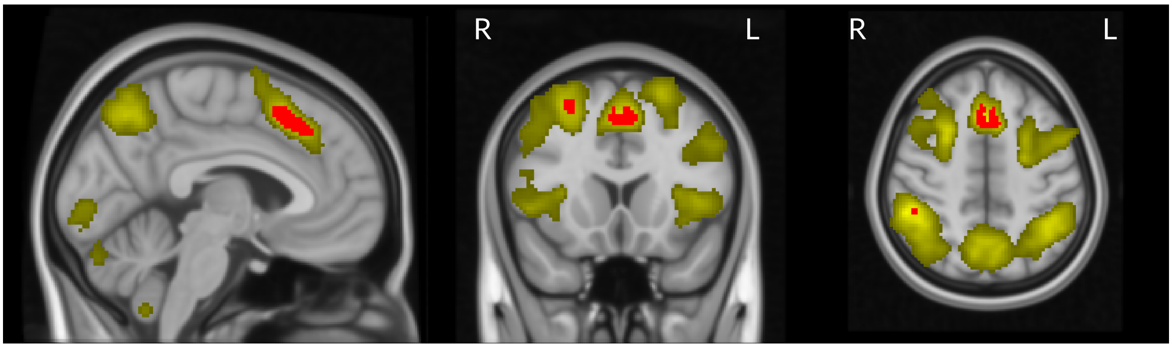		

Supplementary Figure 2: Comparing the upper Confidences Sets for the HCP working memory task data (same slice views as Fig. 16) with the thresholded t-statistic results obtained by applying a traditional group-level one-sample t-test, voxelwise $p < 0.05$ FWE correction (green-yellow voxels). While one large statistically significant cluster covers the supramarginal gyrus, angular gyrus and precuneous, the CSs localize the precise areas with practically significant effect sizes within each of these regions.

Supplementary Table 1. Empirical coverage results for the 2D simulations using nominal (nom.) coverage levels $1 - \alpha = 80\%, 90\%$ and 95%. Results are shown for applying the Wild t-Bootstrap method to the residual field along the estimated boundary $\partial\hat{\mathcal{A}}_c$ (top) and the true boundary $\partial\mathcal{A}_c$ (bottom).

	**2D Signal 1. (Ramp)**		**2D Signal 2. (Circle)**	
	**Standard Dev 1.**	**Standard Dev 2.**	**Standard Dev 1.**	**Standard Dev 2.**
$\partial\hat{\mathcal{A}}_c$				
80% nom.				
$N =$ 60	90.13% ± 0.54%	87.57% ± 0.60%	78.13% ± 0.75%	80.23% ± 0.73%
120	87.53% ± 0.60%	88.40% ± 0.58%	80.53% ± 0.72%	78.70% ± 0.75%
240	87.43% ± 0.61%	87.33% ± 0.61%	79.73% ± 0.73%	79.53% ± 0.74%
480	87.40% ± 0.61%	85.07% ± 0.65%	78.50% ± 0.75%	77.40% ± 0.76%
90% nom.				
$N =$ 60	95.53% ± 0.38%	94.83% ± 0.40%	88.90% ± 0.57%	89.90% ± 0.55%
120	94.07% ± 0.43%	93.73% ± 0.44%	90.13% ± 0.54%	89.40% ± 0.56%
240	94.23% ± 0.43%	93.60% ± 0.45%	89.17% ± 0.57%	90.17% ± 0.54%
480	93.50% ± 0.45%	93.33% ± 0.46%	89.30% ± 0.56%	88.40% ± 0.58%
95% nom.				
$N =$ 60	97.67% ± 0.28%	97.33% ± 0.29%	94.10% ± 0.43%	94.60% ± 0.41%
120	97.13% ± 0.30%	96.60% ± 0.33%	94.40% ± 0.42%	94.37% ± 0.42%
240	97.30% ± 0.30%	97.07% ± 0.31%	94.43% ± 0.42%	95.53% ± 0.38%
480	96.97% ± 0.31%	97.13% ± 0.30%	94.80% ± 0.41%	93.73% ± 0.44%
$\partial\mathcal{A}_c$				
80% nom.				
$N =$ 60	60.27% ± 0.89%	57.30% ± 0.90%	78.17% ± 0.75%	80.23% ± 0.73%
120	66.03% ± 0.86%	68.30% ± 0.85%	80.53% ± 0.72%	78.67% ± 0.75%
240	71.10% ± 0.83%	72.23% ± 0.82%	79.83% ± 0.73%	79.57% ± 0.74%
480	76.27% ± 0.78%	76.17% ± 0.78%	78.57% ± 0.75%	77.40% ± 0.76%
90% nom.				
$N =$ 60	78.47% ± 0.75%	76.60% ± 0.77%	88.97% ± 0.57%	90.00% ± 0.55%
120	81.67% ± 0.71%	83.40% ± 0.68%	90.20% ± 0.54%	89.43% ± 0.56%
240	85.20% ± 0.65%	85.83% ± 0.64%	89.17% ± 0.57%	90.17% ± 0.54%
480	88.50% ± 0.58%	87.23% ± 0.61%	89.27% ± 0.57%	88.43% ± 0.58%
95% nom.				
$N =$ 60	88.97% ± 0.57%	87.27% ± 0.61%	94.17% ± 0.43%	94.57% ± 0.41%
120	89.87% ± 0.55%	90.67% ± 0.53%	94.47% ± 0.42%	94.30% ± 0.42%
240	92.07% ± 0.49%	92.47% ± 0.48%	94.40% ± 0.42%	95.50% ± 0.39%
480	94.23% ± 0.43%	94.10% ± 0.43 %	94.87% ± 0.40%	93.73% ± 0.44%

Supplementary Table 2. Empirical coverage results for the 3D simulations using nominal (nom.) coverage levels $1 - \alpha = 80\%, 90\%$ and 95%. Results are shown for applying the Wild t-Bootstrap method to the residual field along the estimated boundary $\partial\hat{\mathcal{A}}_c$ (top) and the true boundary $\partial\mathcal{A}_c$ (bottom).

	**3D Signal 1. (Small Sphere)**		**3D Signal 2. (Large Sphere)**	
	**Standard Dev 1.**	**Standard Dev 2.**	**Standard Dev 1.**	**Standard Dev 2.**
$\partial\hat{\mathcal{A}}_c$				
80% nom.				
$N =$ 60	83.40% ± 0.68%	83.77% ± 0.67%	85.10% ± 0.65%	85.73% ± 0.64%
120	83.67% ± 0.67%	84.03% ± 0.67%	85.87% ± 0.64%	85.23% ± 0.65%
240	84.03% ± 0.67%	83.77% ± 0.67%	85.23% ± 0.65%	85.40% ± 0.64%
480	85.03% ± 0.65%	82.20% ± 0.70%	87.67% ± 0.60%	85.30% ± 0.65%
90% nom.				
$N =$ 60	92.30% ± 0.49%	92.87% ± 0.47%	92.40% ± 0.48%	93.47% ± 0.45%
120	92.07% ± 0.49%	91.27% ± 0.52%	93.00% ± 0.47%	93.50% ± 0.45%
240	92.33% ± 0.49%	92.87% ± 0.47%	93.30% ± 0.46%	92.90% ± 0.47%
480	93.03% ± 0.46%	91.53% ± 0.51%	93.50% ± 0.45%	93.47% ± 0.45%
95% nom.				
$N =$ 60	96.87% ± 0.32%	96.83% ± 0.32%	96.40% ± 0.34%	96.70% ± 0.33%
120	96.07% ± 0.35%	95.60% ± 0.37%	96.97% ± 0.31%	97.10% ± 0.31%
240	96.20% ± 0.35%	96.83% ± 0.32%	96.23% ± 0.35%	96.90% ± 0.32%
480	96.30% ± 0.34%	96.13% ± 0.35%	96.83% ± 0.32%	93.80% ± 0.44%
$\partial\mathcal{A}_c$				
80% nom.				
$N =$ 60	83.60% ± 0.68%	83.90% ± 0.67%	85.20% ± 0.65%	85.80% ± 0.64%
120	83.80% ± 0.67%	83.93% ± 0.67%	85.90% ± 0.64%	85.23% ± 0.65%
240	84.03% ± 0.67%	83.90% ± 0.67%	85.27% ± 0.65%	85.40% ± 0.64%
480	85.03% ± 0.65%	82.27% ± 0.70%	87.73% ± 0.60%	85.37% ± 0.65%
90% nom.				
$N =$ 60	92.43% ± 0.48%	92.90% ± 0.47%	92.37% ± 0.48%	93.40% ± 0.45%
120	91.97% ± 0.50%	91.43% ± 0.51%	92.97% ± 0.47%	93.60% ± 0.45%
240	92.37% ± 0.48%	92.90% ± 0.47%	93.33% ± 0.46%	92.90% ± 0.47%
480	93.03% ± 0.46%	91.40% ± 0.51%	93.57% ± 0.45%	93.47% ± 045%
95% nom.				
$N =$ 60	96.87% ± 0.32%	96.93% ± 0.31%	96.37% ± 0.34%	96.70% ± 0.33%
120	96.07% ± 0.35%	95.53% ± 0.38%	96.97% ± 0.31%	97.13% ± 0.30%
240	96.17% ± 0.35%	96.93% ± 0.31%	96.23% ± 0.35%	96.80% ± 0.32%
480	96.33% ± 0.34%	96.13% ± 0.35%	96.77% ± 0.32%	96.80% ± 0.32%

Supplementary Table 2. (continued)

	3D Signal 3. (Multiple Spheres)		3D Signal 4. (UK Biobank)
	**Standard Dev 1.**	**Standard Dev 2.**	**UK Biobank SD**
$\partial\hat{\mathcal{A}}_c$			
80% nom.			
$N =$ 60	89.47% ± 0.56%	89.20% ± 0.57%	89.17% ± 0.57%
120	87.60% ± 0.60%	88.17% ± 0.59%	87.17% ± 0.61%
240	86.17% ± 0.63%	86.33% ± 0.63%	86.27% ± 0.63%
480	86.13% ± 0.63%	86.10% ± 0.63%	87.67% ± 0.60%
90% nom.			
$N =$ 60	95.20% ± 0.39%	94.87% ± 0.40%	95.23% ± 0.39%
120	94.53% ± 0.42%	93.97% ± 0.43%	94.63% ± 0.41%
240	93.67% ± 0.44%	93.17% ± 0.46%	93.73% ± 0.44%
480	93.97% ± 0.43%	93.87% ± 0.44%	93.50% ± 0.45%
95% nom.			
$N =$ 60	97.93% ± 0.26%	97.73% ± 0.27%	97.37% ± 0.29%
120	97.37% ± 0.29%	97.47% ± 0.29%	97.73% ± 0.27%
240	97.23% ± 0.30%	96.50% ± 0.34%	96.93% ± 0.31%
480	97.23% ± 0.30%	97.63% ± 0.28%	96.83% ± 0.32%
$\partial\mathcal{A}_c$			
80% nom.			
$N =$ 60	84.30% ± 0.66%	85.33% ± 0.65%	83.30% ± 0.68%
120	84.93% ± 0.65%	86.20% ± 0.63%	85.13% ± 0.65%
240	85.73% ± 0.64%	85.60% ± 0.64%	84.97% ± 0.65%
480	86.03% ± 0.63%	85.97% ± 0.63%	87.73% ± 0.60%
90% nom.			
$N =$ 60	92.93% ± 0.47%	92.20% ± 0.49%	92.77% ± 0.47%
120	93.20% ± 0.46%	93.27% ± 0.46%	93.50% ± 0.45%
240	93.37% ± 0.45%	93.07% ± 0.46%	92.67% ± 0.48%
480	93.97% ± 0.43%	93.80% ± 0.44%	93.57% ± 0.45%
95% nom.			
$N =$ 60	96.80% ± 0.32%	96.50% ± 0.34%	96.70% ± 0.33%
120	96.90% ± 0.32%	96.83% ± 0.32%	97.07% ± 0.31%
240	97.20% ± 0.30%	96.30% ± 0.34%	96.40% ± 0.34%
480	97.27% ± 0.30%	97.63% ± 0.28%	96.77% ± 0.32%
